# Implementation of a Physiologically Based Pharmacokinetic Modeling Approach to Guide Optimal Dosing Regimens for Imatinib and Potential Drug Interactions in Paediatrics

**DOI:** 10.3389/fphar.2019.01672

**Published:** 2020-01-30

**Authors:** Jeffry Adiwidjaja, Alan V. Boddy, Andrew J. McLachlan

**Affiliations:** ^1^ Sydney Pharmacy School, The University of Sydney, Sydney, NSW, Australia; ^2^ School of Pharmacy and Medical Sciences, University of South Australia, Adelaide, SA, Australia; ^3^ University of South Australia Cancer Research Institute, University of South Australia, Adelaide, SA, Australia

**Keywords:** imatinib, physiologically based pharmacokinetic (PBPK), simulation, paediatrics, drug interactions

## Abstract

Long-term use of imatinib is effective and well-tolerated in children with chronic myeloid leukaemia (CML) yet defining an optimal dosing regimen for imatinib in younger patients is a challenge. The potential interactions between imatinib and coadministered drugs in this “special” population also remains largely unexplored. This study implements a physiologically based pharmacokinetic (PBPK) modeling approach to investigate optimal dosing regimens and potential drug interactions with imatinib in the paediatric population. A PBPK model for imatinib was developed in the Simcyp Simulator (version 17) utilizing *in silico*, *in vitro* drug metabolism, and *in vivo* pharmacokinetic data and verified using an independent set of published clinical pharmacokinetic data. The model was then extrapolated to children and adolescents (aged 2–18 years) by incorporating developmental changes in organ size and maturation of drug-metabolising enzymes and plasma protein responsible for imatinib disposition. The PBPK model described imatinib pharmacokinetics in adult and paediatric populations and predicted drug interaction with carbamazepine, a cytochrome P450 (CYP)3A4 and 2C8 inducer, with a good accuracy (evaluated by visual inspections of the simulation results and predicted pharmacokinetic parameters that were within 1.25-fold of the clinically observed values). The PBPK simulation suggests that the optimal dosing regimen range for imatinib is 230–340 mg/m^2^/d in paediatrics, which is supported by the recommended initial dose for treatment of childhood CML. The simulations also highlighted that children and adults being treated with imatinib have similar vulnerability to CYP modulations. A PBPK model for imatinib was successfully developed with an excellent performance in predicting imatinib pharmacokinetics across age groups. This PBPK model is beneficial to guide optimal dosing regimens for imatinib and predict drug interactions with CYP modulators in the paediatric population.

## Introduction

Imatinib has revolutionised the treatment for cancer and led to a subsequent discovery of a class of drugs known as small molecule kinase inhibitors (Rowland et al., 2017). It is approved as the first-line treatment for chronic myeloid leukaemia (CML) and gastrointestinal stromal tumours (GIST) in adult patients and for CML and Philadelphia chromosome-positive (Ph+) acute lymphoblastic leukaemia (ALL) in children and adolescents (Suttorp et al., 2018a). A phase III clinical trial highlighted that imatinib was well tolerated and effective for newly diagnosed paediatric CML, with a 5-year progression free survival of 94% (Suttorp et al., 2018b). A 5-year follow-up of imatinib (340 mg/m^2^/d) in combination with conventional chemotherapy drugs (e.g., cyclophosphamide, methotrexate, and cytarabine) showed a favorable outcome in children with Ph+ ALL, similar to that of bone marrow transplantation (Schultz et al., 2014).

The prevalence of childhood CML and Ph+ ALL, however, is very low, accounting for around 2% of all leukaemias and 3%–5% of ALL in children, respectively (Coebergh et al., 2006). Therefore, an optimal dose for imatinib in paediatric patients, let alone its potential drug-drug interactions, has been less widely explored. Imatinib is mainly metabolised by cytochrome P450 (CYP)3A4 and CYP2C8 (Barratt and Somogyi, 2017), and thus, has a potential for drug interactions with modulators of these CYP enzymes. A clinically significant interaction between imatinib and carbamazepine, a CYP3A and CYP2C8 inducer, was described in a 12-year old CML patient with epilepsy (Taguchi et al., 2014). However, little is known about imatinib interactions with other potential perpetrator drugs in paediatric patients. Conducting a dedicated clinical interaction study in paediatric population remains challenging owing to the ethical and logistical constraints (Barker et al., 2018). Clearly, a feasible and systematic approach to address this gap is warranted.

Physiologically based pharmacokinetic (PBPK) modeling can account for anatomical and physiological growth and organ maturation underlying age-related changes in the pharmacokinetics of a drug of interest (Yellepeddi et al., 2019). This facilitates an extrapolation across the age spectrum (Kuepfer et al., 2016). The PBPK approach has been increasingly embraced by regulatory authorities for the purposes of informing dose selection, providing simulation-based trial design and investigating potential drug interactions in paediatric populations (Cole et al., 2017; Bi et al., 2019). According to applications related to PBPK that were submitted to the US Food and Drug Administration (FDA) from 2008 to 2017, PBPK analyses are mainly intended for evaluating and predicting enzyme-based drug interactions (60% of all applications), followed by utilization in paediatric area (15%) (Grimstein et al., 2019). PBPK modeling and simulation has been an integral part of drug development for paediatric cancers (Rioux and Waters, 2016). PBPK models which can capture developmental changes in biological components are useful in describing in paediatrics the pharmacokinetics of anticancer drugs, including etoposide (Kersting et al., 2012), busulfan (Diestelhorst et al., 2014), docetaxel (Thai et al., 2015), actinomycin D (Walsh et al., 2016) and nilotinib (Heimbach et al., 2019).

A PBPK model for imatinib that incorporates maturational changes of key drug-metabolising enzymes and age-dependent organ development can help inform optimal dose selection in children. PBPK modeling and simulation also provides a greater understanding of potential drug interactions with imatinib in this vulnerable patient group which remains largely unexplored. The aim of this study was to develop and implement a paediatric PBPK model of imatinib for investigating optimal dosing regimens in children and the vulnerability to drug interactions relative to adults with a range of CYP3A modulators.

## Methods

**Figure 1:**
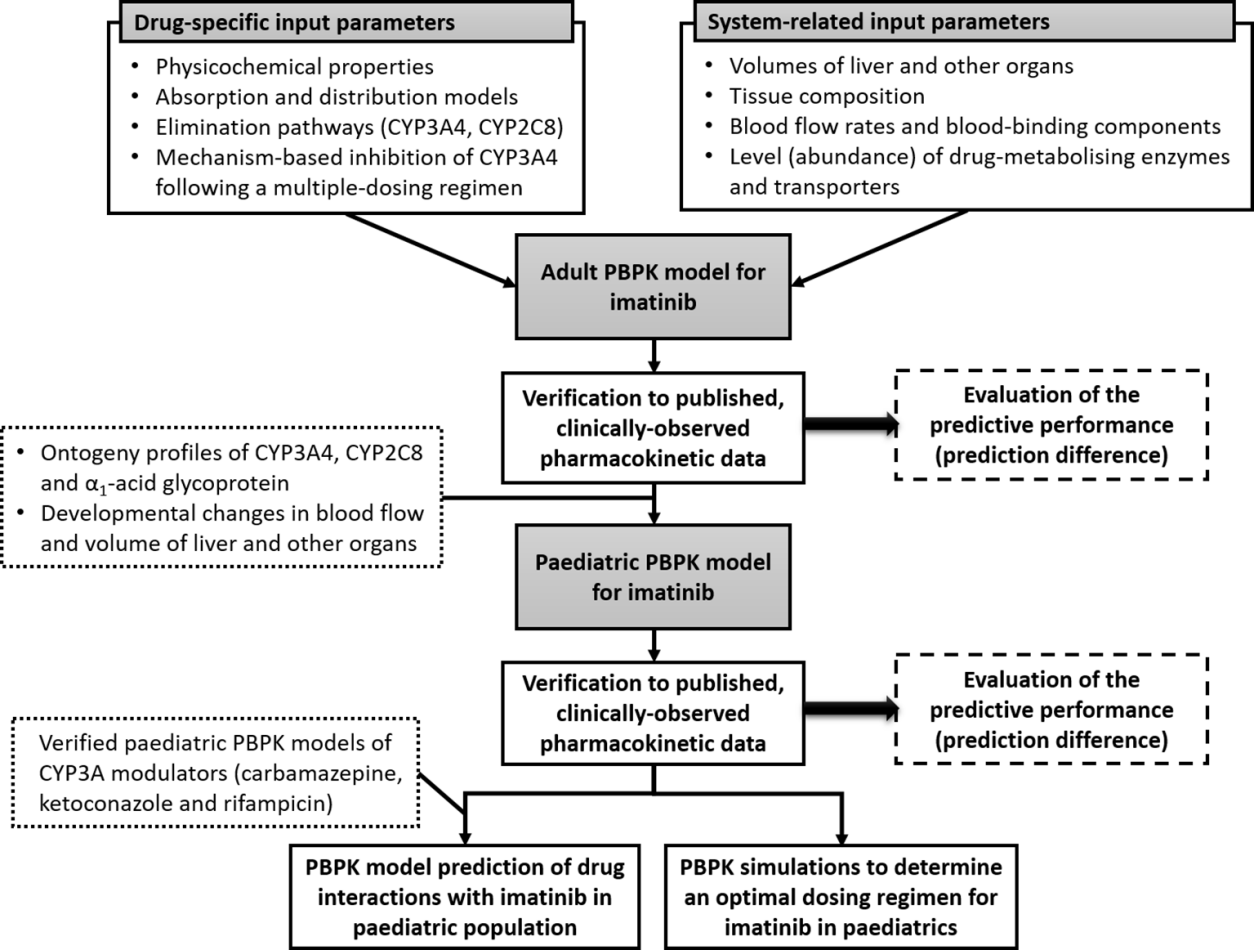
Schematic representation of workflow of this study. Physiologically based pharmacokinetic (PBPK) model of imatinib in adults was constructed using drug-dependent and system-related input parameters and verified using published clinical pharmacokinetic data. The verified model was subsequently extrapolated to children and adolescents by incorporating age-related changes in organ size and maturation of cytochrome P450 (CYP)3A4 and CYP2C8 and α_1_-acid glycoprotein and then verified to clinically observed concentrations in paediatric population. Paediatric PBPK model of imatinib was implemented to determine an optimal dosing regimen for imatinib and evaluate potential drug interactions with a range of CYP3A modulators in children older than 2 years.

In this study, a PBPK model for imatinib was developed and verified in adults and subsequently extrapolated to children and adolescents (aged 2–18 years). The verified PBPK model was then implemented to explore optimal dosing regimens for imatinib in children and to evaluate potential drug interactions with CYP3A modulators. The workflow of this study is summarized in **Figure 1**.

### Development and Verification of a PBPK Model for Imatinib in Adults

All population-based PBPK modeling and simulations were conducted using the Simcyp Simulator (version 17 release 1, Certara UK Limited, Simcyp Division, Sheffield, UK) using the “general North European Caucasian” population library data, which represents typical healthy adult people from European ancestry. The description of Simcyp Simulator workflow, basic algorithm, and ordinary differential equations have been detailed previously (Rowland-Yeo et al., 2010; Jamei et al., 2013). The drug-related input parameters for imatinib are listed in **Table 1**.

**Table 1:** Drug-related parameters used to build a physiologically based pharmacokinetic (PBPK) model for imatinib in Simcyp Simulator.

Parameter	Value	Source
**Physicochemical and blood-binding properties**		
Molecular weight	493.6	PubChem^a)^
Log P_o:w_	1.99	(Peng et al., 2005)
Ionisation pattern	Diprotic base	PubChem and ChEMBL^b)^
pKa	8.07; 3.73	
B/P	0.73	(Kretz et al., 2004)
fu_p_	0.05	(Smith et al., 2004)
Plasma binding component	α_1_-acid-glycoprotein	
**Absorption phase**		
Model	ADAM model	(Darwich et al., 2010)
P_eff_ (10^-4^ cm.s^-1^)	0.92	Predicted in Simcyp Simulator
fu_G_	1	Assumed (Yang et al., 2007)
Q_Gut_ (L.h^-1^)	6.04	Predicted in Simcyp Simulator
**Distribution phase**		
Prediction method	Rodgers and Rowland method	(Rodgers and Rowland, 2007)
V_ss_ (L.kg^-1^)	1.8	Predicted in Simcyp Simulator
**Elimination phase**		
Pathway 1	CYP3A4 (NDMI formation)	
V_max_ (pmol.min^-1^.pmol CYP^-1^)	3.0	Estimated from an *in vitro* study in recombinant CYP3A4
K_m_ (µmol.L^-1^)	10.54	
fu_inc_	0.96	Predicted in Simcyp Simulator
ISEF	0.21	(Chen et al., 2011)
Pathway 2	CYP2C8 (NDMI formation)	
V_max_ (pmol.min^-1^.mg protein^-1^)	56.4	*In vitro* study in HLM of which CYP3A4 enzyme was inactivated by azamulin
K_m_ (µmol.L^-1^)	7.49	
fu_inc_	0.97	Predicted in Simcyp Simulator
Pathway 3	CYP3A4 (other metabolites)	
CL_int_ (µl.min^-1^.mg protein^-1^)	33.4	Estimated from imatinib depletion in recombinant CYP3A4
fu_inc_	1	
Pathway 4	CYP2C8 (other metabolites)	
CL_int_ (µl.min^-1^.mg protein^-1^)	24.2	Calculated from subtraction of *in vivo* CL/F (Widmer et al., 2006) to the sum of scaled CL_int_ from other pathways
fu_inc_	1	
CL_R_ (L.h^-1^)	0.5	(Bornhauser et al., 2005)
Additional HLM CL_int_ (µl.min^-1^.mg protein^-1^)	31	Compensatory clearance for autoinhibition of CYP3A4 at steady-state
**Drug transport – hepatobiliary transporters**		
Pathway 1	ABCB1	
CL_int,T_ (µl.min^-1^.million cells^-1^)	1.5	Calculated from P_eff_ data in ABCB1-transfected MDCK II cells (Dai et al., 2003)
RAF	1	
Pathway 2	ABCG2	
J_max_ (pmol.min^-1^.million cells^-1^)	89.4	Estimated from *in vitro* transport data (Breedveld et al., 2005)
K_m_ (µmol.L^-1^)	4.37	
RAF	0.38	Estimated from *in vivo* biliary clearance of imatinib (Gschwind et al., 2005)
CL_PD_ (ml.min^-1^.million hepatocytes^-1^)	0.2	Assumed
**Drug interactions** (for multiple-dosing of imatinib)		
Mechanism-based inhibition		
k_inact,_ _CYP3A_ (h^-1^)	4.29	(Filppula et al., 2012)
K_I_ (µmol.L^-1^)	14.3	
f_u,inc_	0.8	

ABCB1, multidrug resistance protein 1 or p-glycoprotein; ADAM, advanced dissolution, absorption and metabolism; B/P, blood to plasma ratio; CL_int_, hepatic intrinsic clearance; CL_int,T_, transporter-mediated intrinsic clearance; CL_PD_, passive diffusion clearance; CL_R_, renal clearance; fu_inc_, unbound fraction during incubation; fu_G_, unbound fraction in the enterocytes; fu_p_, unbound fraction in plasma; HLM, human liver microsomes; ISEF, intersystem extrapolation factor; J_max_, maximum flux of a substrate across a drug transporter; K_I_, the concentration that provides half of k_inact_; k_inact_, maximum inactivation rate of CYP enzyme; K_m_, substrate concentration giving half of V_max_ or J_max_; Log P_o:w_, the partition coefficient in oil and water; MDCKII, Madine-Darby canine kidney cells; NDMI, N-desmethyl imatinib; P_eff_, the effective intestinal permeability; pKa, negative logarithm of acid dissociation constant; Q_Gut_, the gut blood flow rate; RAF, relative activity factor; V_max_, maximum rate of reaction; V_ss_, volume of distribution at steady-state based on total tissue volumes.

a) Accessed from pubchem.ncbi.nlm.gov.

b) Accessed from ebi.ac.uk/chembl.

As a basic compound, imatinib binds extensively to α_1_-acid glycoprotein (AAG) (Kretz et al., 2004) with an unbound fraction (fu_p_) of 0.05 (Smith et al., 2004). A higher level of AAG has been reported in patients with solid tumours (Thai et al., 2015). However, plasma AAG concentration is similar in healthy people when compared to patients with CML and GIST (mean value of 0.81 vs. 0.79–1.08 and 0.89 g/L, respectively) (Gambacorti-Passerini et al., 2003; Gandia et al., 2013; Haouala et al., 2013; Bins et al., 2017). This corresponded to an unbound fraction in plasma (fu_p_) for imatinib which was not dissimilar, yet highly variable, between healthy people [0.05 (range 0.02–0.10)] and patients with CML [0.03 (range 0.01–0.10)] (Smith et al., 2004; Gandia et al., 2013). Interestingly, AAG concentrations in patients with GIST were relatively stable over a 1-year course of treatment with imatinib (Bins et al., 2017). Thus, a fixed fu_p_ of 0.05 with associated variability was assigned to adult population. There is a paucity of data on AAG concentration in paediatrics with CML. Nevertheless, clinical data in children with Ph+ ALL (n = 4, aged 6–15 years) hinted at a similar AAG concentration (mean ± standard deviation of 0.88 ± 0.39 g/L) (Marangon et al., 2009) with that of healthy adults and adult patients with CML.

The Advanced Dissolution, Absorption and Metabolism (ADAM) model (Darwich et al., 2010) was used to describe imatinib absorption. The effective intestinal permeability (P_eff_) of imatinib was estimated using the apparent permeability data in Caco-2 cell lines (7.9 x 10^-6^ cm/s). P_eff_ was then utilized to predict the gut blood flow rate (Q_Gut_) (Yang et al., 2007). A whole-body PBPK model was used to describe the distribution of imatinib, with tissue-to-plasma partition coefficient (k_p_) values to each of the organs predicted *in silico* (Rodgers and Rowland, 2007).

The intrinsic clearances (CL_int_) of imatinib to N-desmethyl imatinib (NDMI) and other metabolites were estimated from *in vitro* kinetic data using recombinant CYP3A4 (rCYP3A4) and human liver microsomes (HLM, in the presence of azamulin) as detailed in **Table 1** (unpublished). The latter represented the contribution of CYP2C8, since CYP enzymes other than CYP3A4 and CYP2C8 had a very minor contribution (3%) to imatinib metabolism (Filppula et al., 2013a). Biliary clearance (CL_bile_) of imatinib mediated by ABCB1 and ABCG2 transporters was parameterised by CL_int,T_ or J_max_ and K_m_ the values of which were extracted from previous *in vitro* studies (Dai et al., 2003; Breedveld et al., 2005). Relative activity factor (RAF) of ABCG2 transporter was adjusted to 0.38 to give a CL_bile_ of 28% of overall clearance of imatinib (Gschwind et al., 2005). The renal clearance value for imatinib (CL_R_ = 0.5 L/h) was taken from a study in patients with CML and Ph+ ALL (Bornhauser et al., 2005). The CYP3A4-mediated formation clearance of metabolites other than NDMI (CLu_int,others,3A4_) was estimated from subtraction of depletion clearance of imatinib in rCYP3A4 enzyme (CLu_dep,3A4_) to formation clearance of NDMI in rCYP3A4 (CLu_int,NDMI,3A4_) as detailed in **Table 1**. Intersystem extrapolation factor (ISEF) of 0.21 (Chen et al., 2011) was used to correct for differences in intrinsic activity per unit enzyme between rCYP3A4 and HLM. Clearance of imatinib to other metabolites through a CYP2C8-mediated pathway was estimated according to Eq. 1.

(1) $$\begin{array}{l} CLu_{int,others,2C8}=CLu_{int,total}-\\ \left(CLu_{int,bile}+CLu_{int,NDMI,3A4}+CLu_{int,NDMI,2C8}+CLu_{int,others,3A4}\right) \end{array}$$

where CLu_int,total_ was back-calculated from *in vivo* apparent clearance (CL/F = 14.4 L/h) (Widmer et al., 2006) after subtraction of CL_R_ using the well-stirred hepatic model (a retrograde approach) (Rowland-Yeo et al., 2010).

The mechanism-based inhibition (MBI) of CYP3A4 following a chronic use of imatinib was modeled by an enzyme turnover model as follows:

(2) $$\frac{dEnz}{dt}=k_{deg}.Enz_{0}-k_{deg}.Enz_{\left(t\right)}\left(1+\frac{k_{inact}.fu.\left[I\right]}{K_{Iu}+fu.\left[I\right]}\right);\;Enz_{\left(0\right)}=Enz_{0}$$

where Enz_0_ and Enz_(t)_ indicate the amount of CYP3A4 (Enz) at baseline as reported previously (Cubitt et al., 2011) and at time t, respectively; k_deg_ represents the first-order degradation (turnover) rate constant of the enzyme in hepatocytes and enterocytes (Yang et al., 2008); k_inact_ denotes the maximum rate of inactivation, while K_Iu_ is imatinib concentration needed to reach half of k_inact_, both of which were obtained from a previous report (Filppula et al., 2012); [I] and fu indicate imatinib concentrations in the liver or gut at time t and the unbound fraction of imatinib at the corresponding site of enzyme, respectively. Not accounting for CYP3A4 autoinhibition by imatinib at steady-state led to an overestimation of the extent of interaction with ritonavir, a CYP3A inhibitor, as summarized in **Table S1**. PBPK model predictions which incorporated a CYP3A4 MBI (Eq. 2) were consistent with the clinically observed interaction, however, CL/F of imatinib was underestimated (**Table S1**). A nonpathway specific additional clearance was assigned to the PBPK model at steady-state (**Table 1**) to correct this underprediction. This was also supported by a lack of significant changes in imatinib CL/F at steady-state compared to that on day 1 (Petain et al., 2008; Gotta et al., 2013).

The importance of uptake transporter(s) has been hypothesized since imatinib is almost completely bioavailable, despite being a substrate of both ABCB1 and ABCG2 transporters (Barratt and Somogyi, 2017). The activity of this uptake transporter seems to be diminished by coadministration of gemfibrozil (Filppula et al., 2013b) and in patients who had undergone major gastrectomy (Lubberman et al., 2017). However, available clinical evidence has been conflicting as to which transporter is primarily responsible for the uptake of imatinib (Neul et al., 2016; Barratt and Somogyi, 2017). Coadministration of rifampicin, an inducer and inhibitor of CYP enzymes and SLCO1B transporters, respectively (Kalliokoski and Niemi, 2009; Asaumi et al., 2018) at 600 mg/d for 7 days decreased systemic exposure (AUC_0-∞_) of imatinib given as a single 400 mg oral dose by 74% in healthy adults (Bolton et al., 2004). This suggests that either the uptake process into the liver is not the rate-limiting step for hepatic metabolism of imatinib or sinusoidal uptake transporter(s) other than SLCO1B may play a role. However, the latter is unlikely given that clinical evidence of transporter-mediated drug interactions with imatinib as a victim drug is lacking. Therefore, transporter-mediated uptake processes in gut and liver were not included in the PBPK model.

PBPK simulations of imatinib in adults were performed with trial designs (number of people, age range, proportion of male/female, and dosing regimens) matched to the corresponding clinical studies (**Table 2**). A total of 10 virtual trials for each simulation were carried out. Clinically observed concentrations of imatinib were retrieved from the original publications using WebPlotDigitizer version 4.1 (www.automeris.io/WebPlotDigitizer) and superimposed to simulated profiles to allow visual inspection of the predictive performance. Prediction differences of imatinib pharmacokinetic parameters, expressed as the ratio of PBPK model prediction to clinically reported parameter values were also evaluated.

**Table 2:** Summary of clinical cohorts used for physiologically based pharmacokinetic (PBPK) model verification and comparison of simulated and clinically reported values for pharmacokinetic parameters of imatinib.

Age range (years)	Population	Dosing regimens	Pharmacokinetic parameter	PBPK model prediction^a)^	Clinically observed value	Prediction fold-difference	Reference
**Adult population**							
40–58	Healthy people (n = 12; 2 female)	400 mg, single-dose	C_max_ (µg/ml)	1.6	1.8 ± 1.2	0.89	(Peng et al., 2004)
			t_max_ (h)	2.6	2.5 (1.0–6.0)	1.04	
			AUC_0-∞_ (µg.h/ml)	32.1	32.6 ± 16.5	0.98	
			CL/F (L/h)	12.5	14.9 ± 7.5	0.84	
28–84	Patients with GIST (n = 34; 6 female)	400 mg, day 1	CL/F (L/h)	11.2	10.9^b)^	1.03	(Petain et al., 2008)
			CV of CL/F (%)	51	19^c)^		
		400 mg/d, steady-state	CL/F (L/h)	10.7	10.9^b)^	0.98	
			CV of CL/F (%)	54	19^c)^		
39–82	Patients with GIST (n = 50; 21 female)	400 mg/d, steady-state ^d)^	CL/F (L/h)	9.6	9.1^b)^	1.05	(Eechoute et al., 2012)
			CV of CL/F (%)	52	50^c)^		
18–77	Patients with PAH (n = 103; 83 female)	400 mg/d, steady-state	CL/F (L/h)	9.8	10.8^b)^	0.91	(Renard et al., 2015)
			CV of CL/F (%)	53	43^c)^		
**Paediatric population**							
2–22^e)^	Patients with GIST (n = 33; 13 female)	340 mg/m^2^, day 1	CL/F (L/h)	7.6	7.8^b)^	0.97	(Petain et al., 2008)
			CV of CL/F (%)	69	19^c)^		
		340 mg/m^2^, steady-state	CL/F (L/h)	6.8	7.8^b)^	0.87	
			CV of CL/F (%)	75	19^c)^		
6–24^e)^	Patients with solid tumours and Ph+ leukaemia (n = 41; 14 female)	440 mg/m^2^, day 1	CL/F (L/h)	10.1	10.8^b)^	0.94	(Menon-Andersen et al., 2009)
			CV of CL/F (%)	63	32^c)^		
		440 mg/m^2^, steady-state	CL/F (L/h)	8.7	10.8^b)^	0.81	
			CV of CL/F (%)	62	32^c)^		
4–17	Patients with CML (n = 26; 6 female)	300 mg/m^2^, steady-state	C_min_ (µg/ml)	1.2	1.4 ± 0.8	0.86	(Jaeger et al., 2012; Suttorp et al., 2018a)
6–15	Patients with Ph+ ALL (n = 4; 2 female)	300 mg/m^2^, day 1	C_max_ (µg/ml)	3.3	3.9 (2.7–5.1)	0.85	(Marangon et al., 2009)
			AUC_24_ (µg.h/ml)	49	55 (37–74)	0.89	
		300 mg/m^2^, steady-state	C_ss,max_ (µg/ml)	4.5	6.1 (3.8–8.4)	0.74	
			AUC_24_ (µg.h/ml)	59	73 (60–87)	0.81	
2–18	Patients with tumours in CNS (n = 4; 1 female)	300 mg bid, day 1 and steady-state	C_max_ (µg/ml)	2.7	2.5 (1.7–3.0)	1.08	(Baruchel et al., 2009)
			C_min_ (µg/ml)	3.9	3.3 (2.1–3.7)	1.18	
	Patients with tumours in CNS (n = 1; no female)	500 mg/d, day 1 and steady-state	C_max_ (µg/ml)	5.4	4.9	1.10	
			C_24_ (µg/ml)	0.9	0.9	1.00	
			C_min_ (µg/ml)	2.1	2.1	1.00	

AUC_0-∞_, area under the plasma concentration-time curve from time zero to infinity; AUC_24_, area under the plasma concentration-time curve during 24 h after dose; C_24_, plasma concentration at 24 h; C_max_, peak plasma concentration; C_min_, trough concentrations; CNS, central nervous system; C_ss,max_, peak plasma concentration at steady-state; CL/F, apparent clearance; CML, chronic myeloid leukaemia; CV, coefficient of variation; GIST, gastrointestinal stromal tumours; PAH, pulmonary arterial hypertension; Ph+ ALL, Philadelphia chromosome-positive acute lymphoblastic leukaemia; t_max_, time required to achieve peak plasma concentration.

a) Reported as geometric mean values of PBPK model prediction.

b) Typical population value.

c) Based on ω (standard deviation of eta, interindividual variability) of apparent clearance.

d) 26% of the cohort received 800 mg/d of imatinib.

e) This cohort also includes young adult patients.

### Extrapolation of the PBPK Model of Imatinib to Paediatric Population

**Figure 2:**
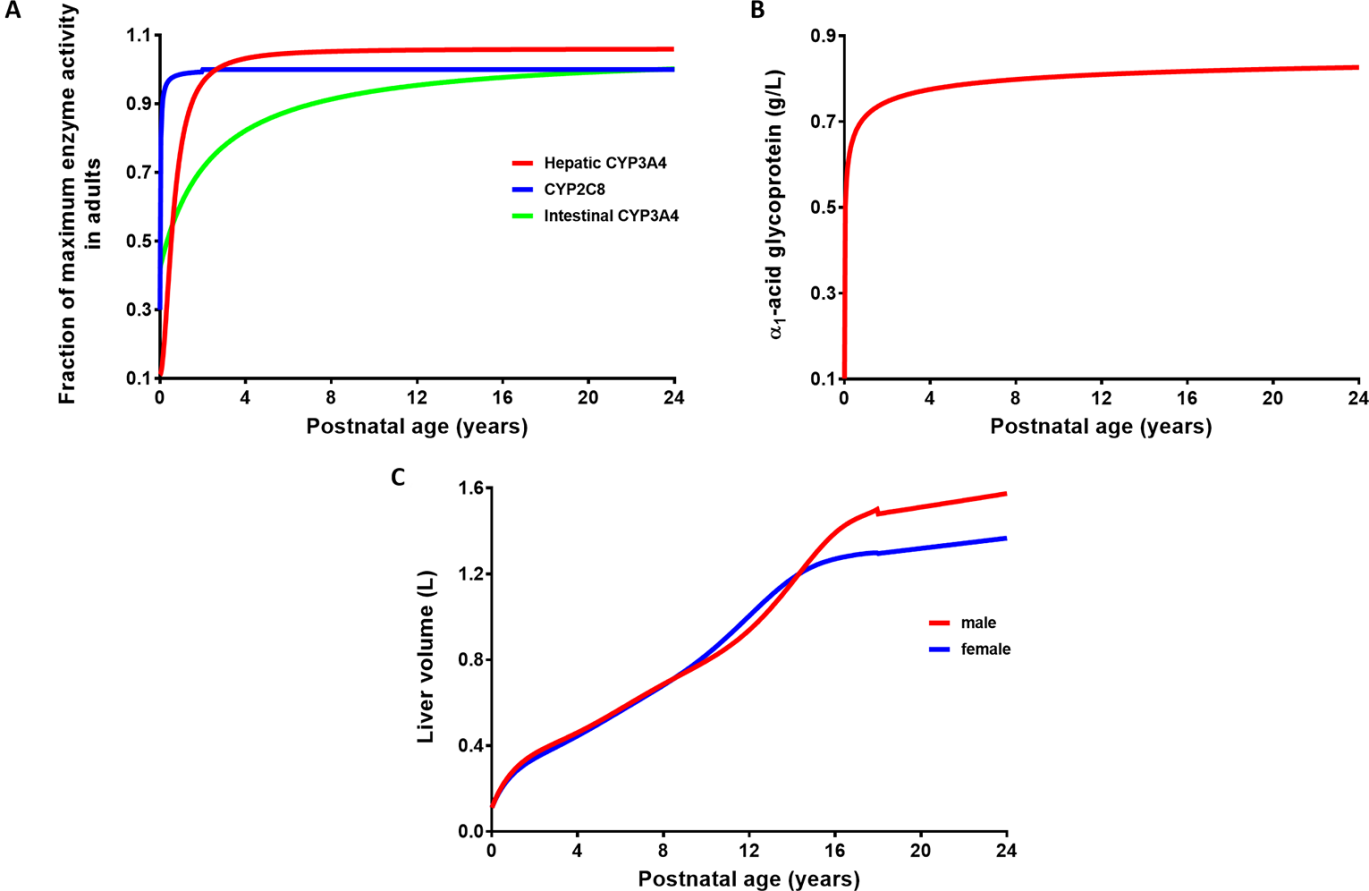
Ontogeny profiles of drug-metabolising enzymes responsible for imatinib metabolism **(A)** and age-related changes in plasma concentration of α_1_-acid glycoprotein **(B)** and liver volume **(C)**.

The verified PBPK model of imatinib in the adult population was extrapolated to children and adolescents (2–18 years) according to the best practice in development of paediatric PBPK model (Maharaj et al., 2013; Maharaj and Edginton, 2014). Drug-specific parameters for imatinib were fixed at the same values as those defined in the adult PBPK model (**Table 1**). The algorithms for ontogeny profiles of CYP enzymes (**Figure 2A**) are incorporated into Simcyp Simulator by default (Johnson et al., 2006). A sigmoidal E_max_ model (Eq. 3), driven by postnatal age, adequately describes the maturation of CYP3A4 and CYP2C8. Parameters specific to each enzyme are summarized in **Table 3**.

(3) $$Fraction\;of\;adult=\;F_{birth}+\frac{(adult_{max}-F_{birth})\times PNA^{n}}{PNA_{50}^{n}+PNA^{n}}$$

**Table 3:** Parameters used in sigmoidal E_max_ functions to describe the maturation of drug-metabolising enzymes involved in imatinib metabolism.

Parameter	Hepatic CYP3A4	Intestinal CYP3A4	Hepatic CYP2C8
Adult_max_	1.06	1.06	1.00
PNA_50_ (years)	0.64	2.36	0.02
F_birth_	0.11	0.42	0.30
n	1.91	1.00	1.00

Adult_max_, maximum fractional level of expression in adults; CYP, cytochrome P450, F_birth_, fraction of CYP enzymes at birth relative to adult; n, the Hill coefficient; PNA_50_, time to reach half of adult_max_.

where adult_max_ represents the maximum level of expression (as a fraction) of CYP enzymes in adult population; F_birth_ is the fraction of CYP enzymes at birth relative to adult; n denotes an exponent which is analogous to the Hill coefficient; PNA and PNA_50_ are postnatal age and the maturation half-life in years, respectively.

The ontogeny function derived for α_1_-acid glycoprotein (AAG) as shown in Eq. 4 and **Figure 2B** was based on a limited set of data compiled from previously published reports (Johnson et al., 2006) and as an update of McNamara and Alcorn’s linear equation (McNamara and Alcorn, 2002). Interestingly, this sigmoidal E_max_ model is very similar to the one generated recently from a larger meta-analysis in healthy people (Maharaj et al., 2018). Unbound fraction of imatinib in paediatrics (fu_ped_) was then estimated based on the ratio of plasma concentrations of AAG to that in the adult population (Eq. 5). Developmental changes in organ blood flow (as percent cardiac output to different organs) and organ size have been detailed previously (Johnson et al., 2006). The changes in liver size with body surface area (BSA) are specified in Eq. 6 (Johnson et al., 2005), where BSA (m^2^) was estimated from body weight and height of each individual (DuBois and DuBois, 1916). The associated changes in liver size based on age and sex are depicted in **Figure 2C**.

(4) $$AAG\;\left(g/L\right)=\;\frac{0.887\times {\left(365\times PNA\right)}^{0.38}}{8.89^{0.38}+{\left(365\times PNA\right)}^{0.38}}$$

(5) $$fu_{ped}=\frac{1}{1+\frac{AAG_{ped}}{AAG_{adult}}.\frac{\left(1-fu_{adult}\right)}{fu_{adult}}}\;$$

(6) $$Liver\;volume\;(L)=\;0.722\times BSA^{1.176}$$

where PNA denotes postnatal age in years; AAG_adult_ and AAG_ped_ are plasma concentrations of AAG in adult and paediatric population, respectively; and fu_adult_ is the unbound fraction of imatinib in adults (mean value of 0.05).

Given the importance of ABCB1 and ABCG2 transporters on biliary excretion of imatinib (Barratt and Somogyi, 2017), the maturation rates of these drug transporters need to be considered. The expression of hepatic and intestinal ABCG2 transporter was not affected by age (Prasad et al., 2016; Cheung et al., 2019), while there are conflicting data on developmental changes in protein expression of hepatobiliary ABCB1 transporter (Mooij et al., 2014; Prasad et al., 2016). However, the clinical pharmacokinetic data and PBPK simulations of digoxin, a probe drug for ABCB1, suggest a rapid maturation and attainment of adult levels of expression within first few months after birth (Johnson et al., 2016). Therefore, no age-related change was assumed for ABCB1 transporter and the adult values, which is the default setting in Simcyp Simulator, were applied.

The PBPK model in paediatrics was verified using published, clinical pharmacokinetic data following single- and multiple-dosing regimens of imatinib. Simulations were performed (10 virtual trials for each simulation) with a trial design similar to the corresponding clinical studies as presented in **Table 2**. It is worth mentioning that the age range of participants in a number of clinical studies overlaps with that of young adults (Petain et al., 2008; Menon-Andersen et al., 2009). However, this was acceptable since all ontogeny functions employed in the model followed a clear trajectory until adult age (**Figure 2**).

### PBPK Simulation to Evaluate Optimal Dosing Regimens for Imatinib in Paediatrics

The paediatric population was categorised into several age groups: preschool (2–5 years) and school-age children (6–11 years), and adolescents (12–17 years) (Batchelor and Marriott, 2013). PBPK simulations of imatinib were performed using hypothetical multiple-dosing regimens given for 14 days (steady-state was assumed to be achieved within this time frame) with n = 100 (40% of female) for each age group. The male-to-female ratio was based on the value observed in paediatric patients, in which boys had an approximately 1.3-fold higher risk to be diagnosed with CML (Coebergh et al., 2006). BSA-normalized doses of imatinib of 170, 230, 340, and 460 mg in paediatrics corresponded to fixed doses of 300, 400, 600, and 800 mg in adults, respectively. The total daily doses of imatinib (in mg) for each age band were rounded to the closest 50 mg, a half-size of the smallest commercially available imatinib tablet as recommended in the clinical setting (Suttorp et al., 2018a) and were capped at the equivalent adult doses. Potential differences of imatinib C_min_ across age bands were evaluated by a one-way analysis of variance (ANOVA) with Tukey post hoc test using GraphPad Prism version 7.02 (GraphPad Software, La Jolla, CA, USA).

### PBPK Model Prediction of Drug Interactions With a Range of CYP3A Modulators

#### Verification of Paediatric PBPK Models for Carbamazepine, Ketoconazole, and Rifampicin

The default PBPK models for carbamazepine, ketoconazole, and rifampicin in Simcyp Simulator were used (Almond et al., 2016; Liu et al., 2017). The predictive performance of the PBPK models in paediatric population need to be verified prior to their further use, since the original models were developed in adults. PBPK simulations for the three CYP3A modulators were carried out across different dosing regimens and age groups as detailed in **Table 4**, with a total of 10 virtual trials for each of the simulations. The predicted fold-differences of pharmacokinetic parameters for each compound, expressed as PBPK model prediction over the values reported in clinical studies were determined.

**Table 4:** Comparison of physiologically based pharmacokinetic (PBPK) model prediction and clinically observed values for pharmacokinetic parameters of carbamazepine, ketoconazole, and rifampicin in paediatric population.

Dosing regimens	Population	Age range (years)	Pharmacokinetic parameter	PBPK model prediction^a)^	Clinically observed value	Prediction fold-difference	Reference
**Carbamazepine**							
300 mg bid, multiple-dose	Patients with epilepsy (n = 52; 21 girls)	2–21	CL/F (L/h)	3.8	3.6^b)^	1.06	(Carlsson et al., 2005)
			CV of CL/F (%)	54	52^c)^		
9.5 mg/kg bid, multiple-dose	Patients with epilepsy (n = 21; 10 girls)	4–13	C_ss,max_ (µmol/L)	40.2	39.8 ± 10.0	1.01	(Eeg-Olofsson et al., 1990)
			C_min_ (µmol/L)	19.0	21.5 ± 5.8	0.88	
			AUC_24_ (µmol.h/L)	742.3	762.5 ± 163.2	0.97	
**Carbamazepine-10,11-epoxide**							
9.5 mg/kg bid of carbamazepine, multiple-dose	Patients with epilepsy (n = 21; 10 girls)	4–13	C_ss,max_ (µmol/L)	5.5	6.0 ± 2.3	0.92	(Eeg-Olofsson et al., 1990)
			C_min_ (µmol/L)	4.5	4.0 ± 1.6	1.13	
			AUC_24_ (µmol.h/L)	121.4	138.0 ± 48.9	0.88	
**Ketoconazole**							
5 mg/kg, single-dose	Patients with oral candidiasis (n = 12; 5 girls)	2–12.5	AUC_6_ (µg.h/ml)	17.5	15.3 ± 2.7	1.14	(Ginsburg et al., 1983)
4.8 mg/kg bid, multiple-dose	Patients with candidiasis (n = 7; 3 girls)	1–14	C_ss,max_ (µg/ml)	4.6	3.5 ± 0.9	1.31	(Bardare et al., 1984)
			AUC_12_ (µg.h/ml)	19.9	13.6 ± 2.4	1.46	
8.7 mg/kg/d, multiple-dose	Patients with candidiasis (n = 4; 1 girl)	1–12	C_ss,max_ (µg/ml)	8.1	6.3 ± 1.7	1.29	(Bardare et al., 1984)
			AUC_24_ (µg.h/ml)	34.9	40.7 ± 8.7	0.86	
**Rifampicin**							
10 mg/kg, single-dose	Patients with impetigo or cellulitis (n = 21; 10 girls)	0.5–5	AUC_8_ (µg.h/ml)	47	56	0.84	(McCracken et al., 1980)
300 mg/m^2^ (30-min i.v. infusion), single-dose	Patients with *H. influenzae* infections (n = 20; 9 girls)	0.25–3	C_max_ (µg/ml)	30.8	27.4 ± 12.1	1.12	(Koup et al., 1986a)
			CL_i.v._ (L/h/m^2^)	4.1	3.7 ± 1.3	1.11	
300 mg/m^2^ tid (30-min i.v. infusion), multiple-dose	Patients with staphylococcal infections (n = 12; 5 girls)	0.25–13	C_ss,max_ (µg/ml)	28.4	25.9 ± 1.3	1.10	(Koup et al., 1986b)
			CL_i.v._ (L/h/m^2^)	4.3	4.0 ± 1.5	1.08	

AUC_6_, AUC_8_, AUC_12_, AUC_24_, area under the plasma concentration-time curve during 6, 8, 12 and 24 h after dose, respectively; bid, twice daily; C_max_, peak plasma concentration; C_min_, trough concentration; C_ss,max_, peak plasma concentration at steady-state; CL_i.v._, clearance after intravenous administration; CL/F, apparent clearance; CV, coefficient of variation; tid, three times a day; i.v., intravenous.

a) Reported as geometric mean values of PBPK model prediction.

b) Typical population value.

c) Based on ω (standard deviation of eta, interindividual variability) of apparent clearance.

#### Evaluation of PBPK Model Prediction of Interaction With Carbamazepine

PBPK simulations were performed to predict the extent of interaction between carbamazepine and imatinib in adults (n = 63, age ranging from 19 to 69 years) (Pursche et al., 2008) and paediatrics (a 12-year old male) (Taguchi et al., 2014). Designs of the clinical studies were replicated in the PBPK simulations, except for the latter which was carried out in a total of 100 subjects with age range of 11–13 years. This was necessary since the Simcyp Simulator does not allow the assignment of a single age value in a trial design. The goodness-of-fit of the PBPK predictions was evaluated via a visual inspection of the simulated pharmacokinetic profiles which were overlaid to imatinib concentrations observed in the clinical studies.

#### Implementation of PBPK Modeling Approach to Evaluate Drug Interactions With CYP3A Modulators Across Different Age Bands

To investigate the age-related changes in liability to CYP modulation, PBPK prediction of imatinib interactions with a set of CYP3A modulators, exemplified by carbamazepine, ketoconazole and rifampicin were conducted in adult and paediatric populations. The verified PBPK models in paediatrics were used, with the addition of CYP2C8 induction to the rifampicin model (maximum fold of induction (Ind_max_) = 6.27 and concentration that provides half of Ind_max_ (IndC_50_) = 0.1 µmol/L) (Raucy et al., 2002). CYP2C8 induction was also incorporated to carbamazepine and its active metabolite, carbamazepine-10,11-epoxide with IndC_50_ and Ind_max_ for both compounds of 22 µmol/L and 3.5, respectively (Zhang et al., 2015). The induction of CYP3A and CYP2C8 was modeled by an increase in protein synthesis (turnover) rate constant in hepatocytes and enterocytes according to an enzyme turnover model (Almond et al., 2016). Ketoconazole inhibits CYP3A4 and CYP2C8 competitively with an inhibitory constant (Ki_u_) of 15 nmol/L (Liu et al., 2017) and 2.2 µmol/L, respectively. PBPK simulations were carried out with n = 50 (40% of female) for each age band. Imatinib was given for 14 days with and without carbamazepine, ketoconazole or rifampicin. Imatinib was administered at a daily dose of 230 mg/m^2^ and 400 mg for paediatrics and adults, respectively. The typical maintenance dosing regimens were assigned for each CYP3A modulator based on age ranges. Potential changes in area under the plasma concentration-time curve (AUC) of imatinib for each age group on the last day was predicted.

## Results

### Development and Verification of a PBPK Model for Imatinib in Adults

**Figure 3:**
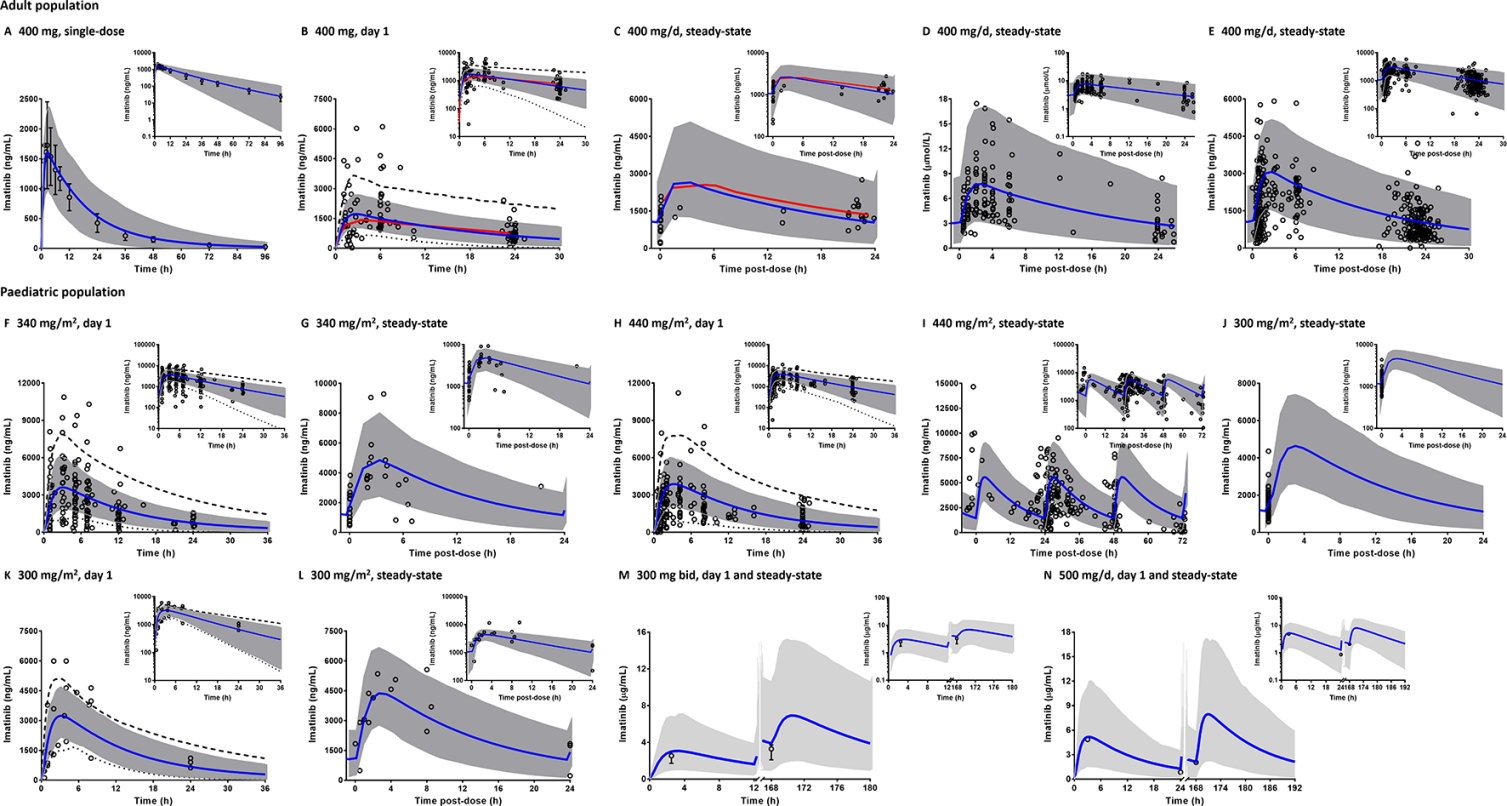
Comparison of physiologically based pharmacokinetic (PBPK) model prediction and clinically observed concentrations of imatinib in adult **(A**–**E)** and paediatric populations **(F**–**N)**. PBPK simulations are presented as mean simulated concentrations (blue line) with their 5^th^ to 95^th^ percentiles (grey area) in linear scale with the corresponding semi-logarithmic plots as insets. Dashed and dotted black lines represent maximum and minimum simulated concentrations, respectively. Clinical pharmacokinetic data (circles) are depicted as either individual data **(B**–**L, N)** or mean concentrations with whiskers as corresponding standard deviations **(A**, **M)**. Population pharmacokinetic predictions of imatinib concentration are shown by red line **(B**, **C)**. Bid, twice a day.

The PBPK model was successfully predicted pharmacokinetic of imatinib following single- and multiple-dosing regimens in adults (**Figures 3A–E**). Clinically observed concentrations of imatinib fell within 5^th^ to 95^th^ percentiles of the PBPK model simulated pharmacokinetic profiles. Interestingly, PBPK simulation of the study by Petain et al. were in close agreement with those predicted using a population pharmacokinetic approach (Petain et al., 2008) as shown in **Figures 3B, C**. However, the observed interindividual variability of imatinib concentrations on day 1 appears to be underestimated (**Figure 3B**). All the key pharmacokinetic parameters of imatinib were predicted within a 1.25-fold difference (range: 0.84–1.05).

### Extrapolation of the PBPK Model of Imatinib to Paediatric Population

PBPK model predictions in paediatrics (2–18 years) were consistent with clinically observed pharmacokinetic data (**Figures 3F–N**), although the interindividual variability of imatinib concentrations following single-doses of 300 (Marangon et al., 2009), 340 (Petain et al., 2008) and 440 mg/m^2^ (Menon-Andersen et al., 2009) appeared to be underpredicted. A number of the clinical pharmacokinetic data came from studies with sparse sampling points, e.g. restricted to imatinib C_min_ (Suttorp et al., 2018a) or only 1–2 samples from few children (Baruchel et al., 2009). However, PBPK simulations were able to capture the overall trend observed in the corresponding clinical studies (**Figures 3J, M, N**). All simulated pharmacokinetic parameters fell within 1.25-fold of those reported in clinical pharmacokinetic studies (**Table 2**), except for peak concentrations of imatinib at steady-state (C_ss,max_) in the study by Marangon et al (2009).

### PBPK Simulation to Evaluate Optimal Dosing Regimens for Imatinib in Paediatrics

**Figure 4:**
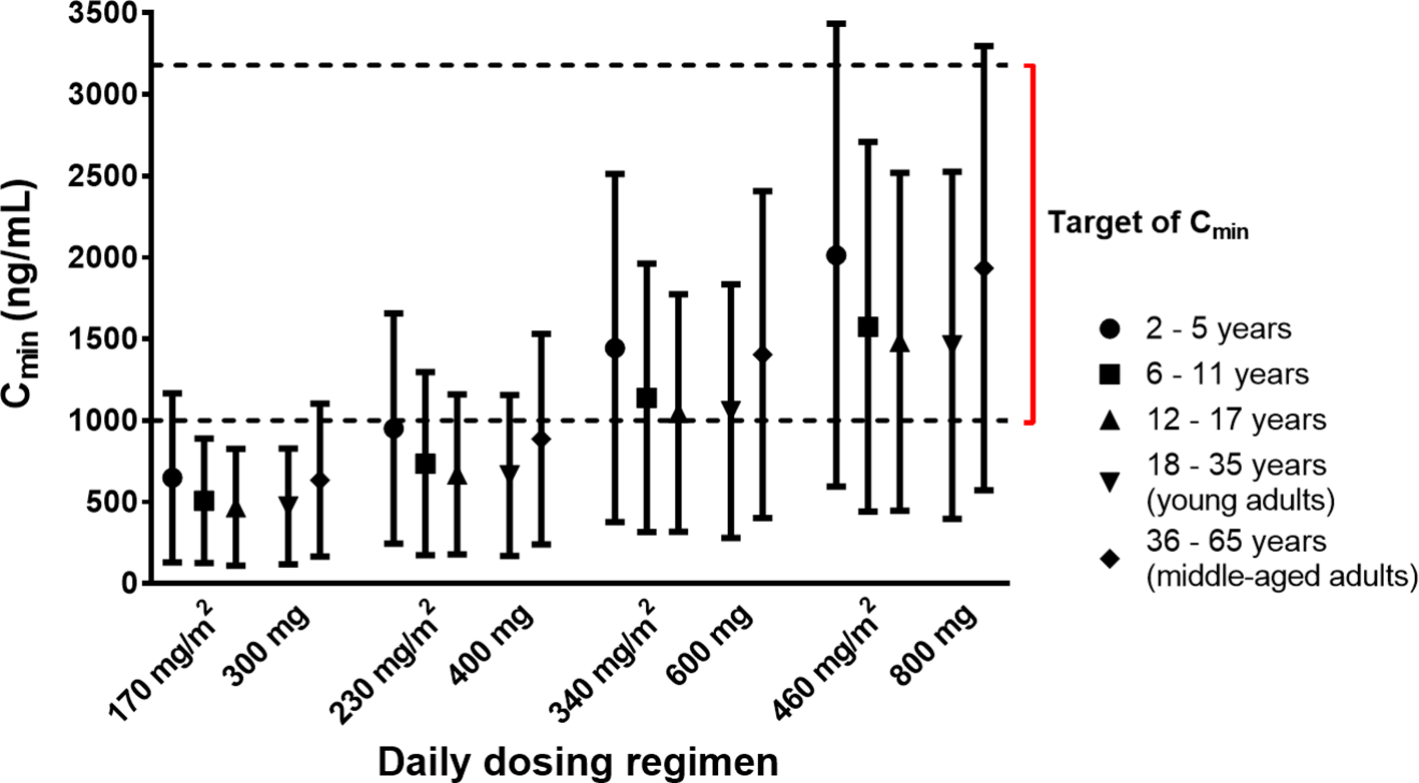
Simulated trough concentrations (C_min_) of imatinib stratified by age bands following various dosing regimens. Simulated data are shown as mean (symbols) with whiskers correspond to standard deviations. The lower and upper limits of target C_min_ (1,000–3,200 ng/ml) are indicated by dashed black lines.

The C_min_ targets of at least 1,000 ng/ml (Larson et al., 2008; Verheijen et al., 2017) and more strictly, between 1,000 and 3,200 ng/ml (Lankheet et al., 2017) were used for the simulations. PBPK simulations indicated that the variability of the attained C_min_ of imatinib was higher in the paediatric population at age 2 to 5 years and middle-aged adults compared to other age groups (**Figure 4**). The mean C_min_ after a daily dose of 340 mg/m^2^ were predicted to be above the target concentration of 1,000 ng/ml irrespective of the age group. At a lower dose (230 mg/m^2^), imatinib C_min_ values were predicted to be lower than the predefined target concentration in a large subset of children above 5 years of age (**Figure 4**). Statistical analysis of C_min_ of imatinib given at a daily dose of 230 and 340 mg/m^2^ in paediatrics (corresponded to 400 and 600 mg in adults, respectively) indicated that there was no significant difference among different age bands *(p* > 0.01).

### PBPK Model Prediction of Drug Interactions With a Range of CYP3A Modulators

**Figure 5:**
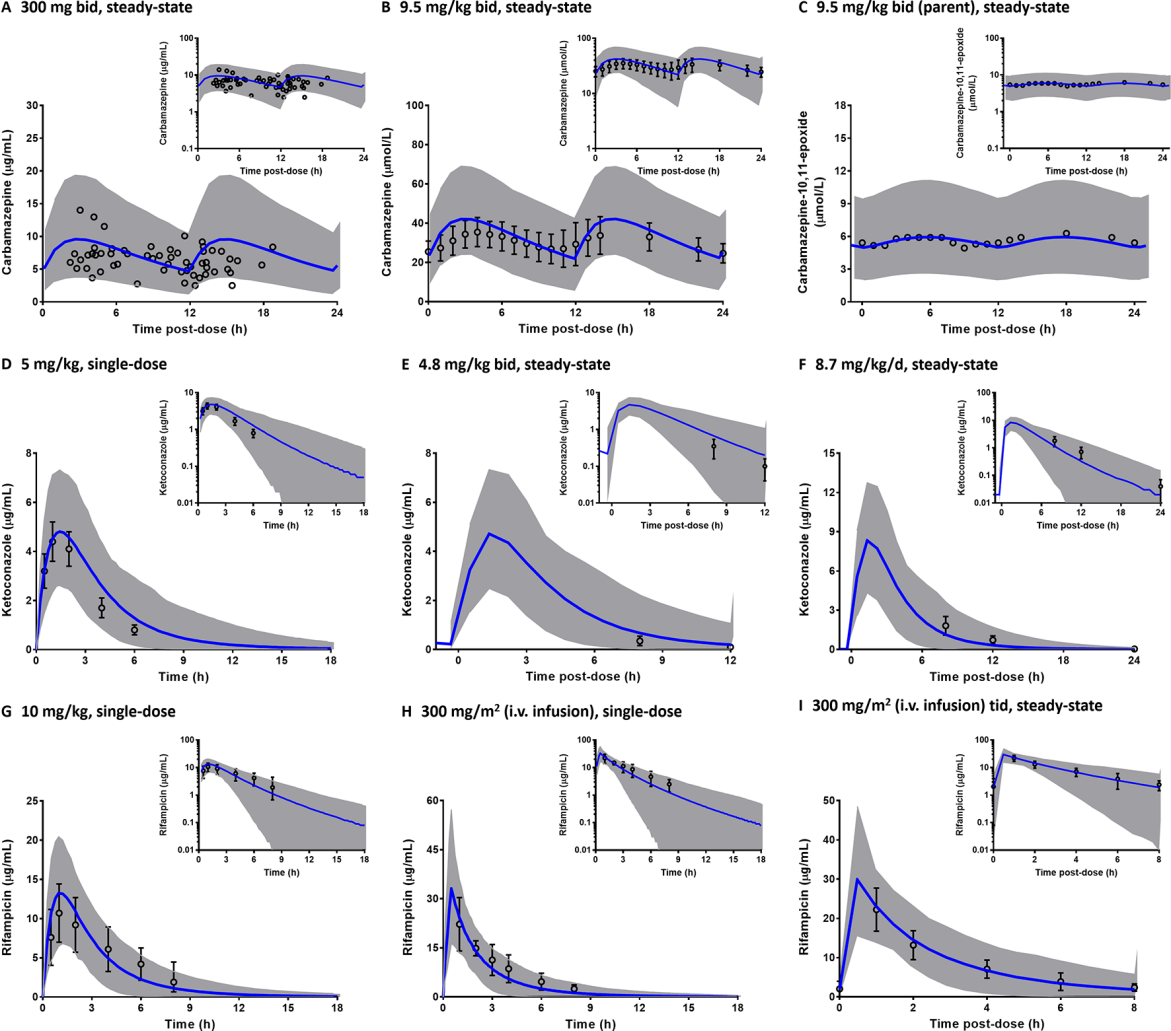
Predicted pharmacokinetic profiles of carbamazepine **(A**–**B)** and its metabolite, carbamazepine-10,11-epoxide **(C)**, ketoconazole **(D**–**F)**; and rifampicin **(G**–**I)** in paediatrics. The predictions are depicted in linear scale with the corresponding semi-logarithmic plots as insets (blue line: mean, grey area: 5^th^ to 95^th^ percentiles). Clinically observed concentrations (circles) are presented either as individual data **(A)**, mean **(C)** or mean with the associated standard deviations **(B**, **D**–**I)**. Tid, three times a day.

**Figure 6:**
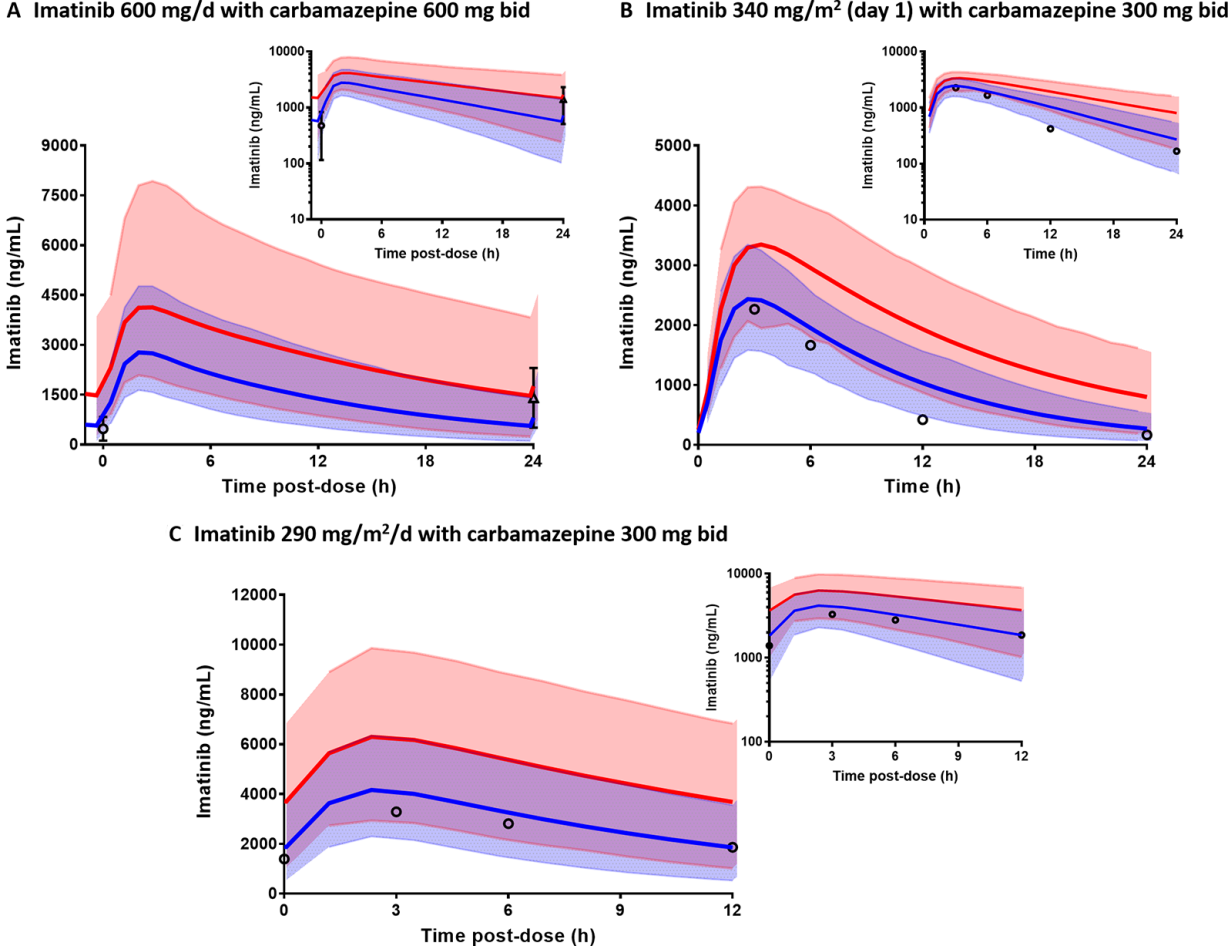
Physiologically based pharmacokinetic (PBPK) model prediction of imatinib concentrations in the presence (blue line) and absence of carbamazepine (red line) in adults **(A)** and paediatric **(B, C)**. Prediction intervals (5^th^ to 95^th^ percentiles) for imatinib concentrations with and without carbamazepine are represented by light blue and pink area, respectively. Clinically observed data are represented by mean concentrations of imatinib alone (triangle) or with carbamazepine (circles) with whiskers as corresponding standard deviations.

Comparisons of the prediction interval (mean concentrations and 5^th^ to 95^th^ percentiles) with the clinically observed pharmacokinetic data for carbamazepine, rifampicin, and ketoconazole at various dosing regimens in paediatrics are presented in **Figure 5**. Carbamazepine is primarily metabolised by CYP3A and CYP2C8 enzymes and thus, induces its own metabolism (Thorn et al., 2011). Interestingly, accounting for CYP2C8 induction in the PBPK model of carbamazepine and its active metabolite (carbamazepine-10,11-epoxide) in paediatrics improved the predictions (**Figures 5A–C**; PBPK simulations without CYP2C8 induction are not shown). Prediction differences for pharmacokinetic parameters of carbamazepine and its metabolite in the presence and absence of CYP2C8 autoinduction were within 1.25-fold (range: 0.88–1.13) and 1.5-fold (range: 0.89–1.45), respectively (**Table S2**). In line with that, the decrease of imatinib C_min_ when coadministered with carbamazepine (**Figure 6A**) was better predicted by the PBPK model that incorporates CYP2C8 induction [C_min_ ratio of 0.38 vs. 0.47, compared to the clinically reported value of 0.34 (Pursche et al., 2008)]. Clinical pharmacokinetic data for the corresponding interaction in paediatrics are sparse, limited to imatinib concentrations from a child on day 1 and at steady-state in the presence of multiple-doses of carbamazepine (Taguchi et al., 2014). Despite that, the verified PBPK model of imatinib in paediatric population described the clinical interaction data with a good accuracy, as shown in **Figures 6B, C**.

**Figure 7:**
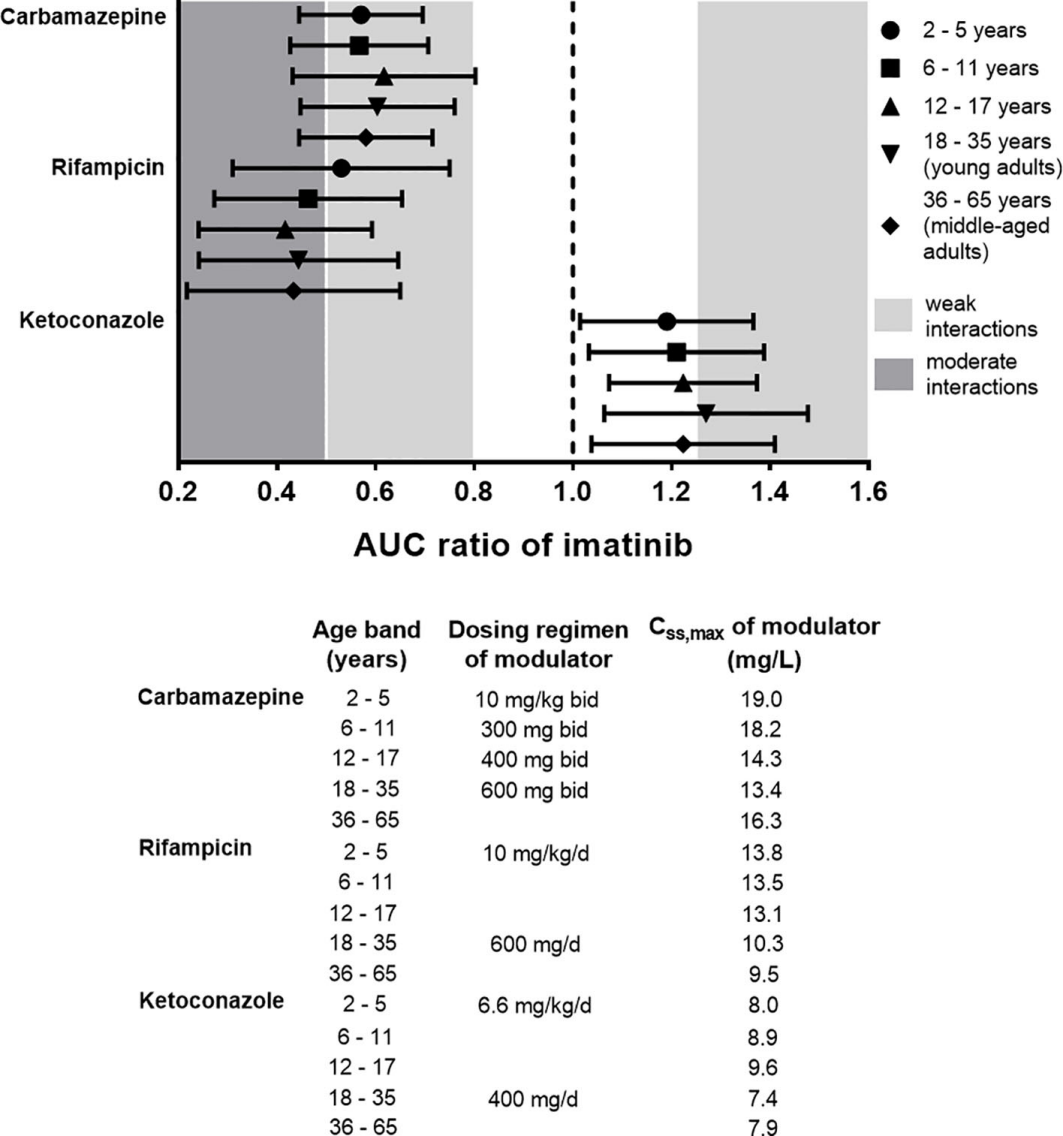
Physiologically based pharmacokinetic (PBPK) prediction of imatinib interactions with a set of CYP3A modulators (carbamazepine, ketoconazole, and rifampicin) at steady-state across different age bands. Imatinib at daily doses of 400 mg and 230 mg/m^2^ was administered to adult and paediatric populations, respectively along with CYP3A modulators for 14 days. The extent of interactions was evaluated based on AUC ratio metric (ratio of area under the plasma concentration-time curve of imatinib in the presence and absence of CYP3A modulators). Symbols represent median simulated AUC ratio with whiskers crossing from 5^th^ to 95^th^ percentiles. C_ss,max_, peak concentration at steady-state. AUC ratio of 1 (dotted black line) indicates absence of drug interactions with imatinib. Typical dosing regimens and the attained C_ss_,_max_ of the modulators for each age band in the PBPK simulations are also detailed.

In addition to carbamazepine, the PBPK model was also implemented for prediction of interactions with ketoconazole and rifampicin across different age groups (2–65 years). Predicted AUC ratios of imatinib in the presence and absence of each of the modulators are summarized in **Figure 7**. It is noteworthy that the administration CYP3A modulators at their typical maintenance dosing regimens according to age bands yielded C_ss,max_ that were comparable across all groups, except for rifampicin, where C_ss,max_ was around 30% lower in middle-aged adults compared to children less than 18 years (**Figure 7**). This was important to evaluate the extent of interactions among different age groups without being confounded by steady-state concentrations of the modulators. Further statistical analysis suggested that there were no significant differences in the extent of interactions between different age bands (one-way ANOVA followed by a Tukey post-hoc analysis, *p* > 0.01).

## Discussion

We developed a PBPK model for imatinib in adult populations and extrapolated its use to paediatrics. The PBPK model was able to describe imatinib pharmacokinetics in both populations and had a capability to predict drug interactions with a range of CYP3A modulators.

A paediatric PBPK model for imatinib has been reported previously in a regulatory document submitted to European Medicines Agency (2013). Unfortunately, a lack of details regarding this PBPK model’s structure and parameters limits its further use and interpretation. The PBPK model in the current study was verified to a larger set of clinically published pharmacokinetic data and its implementation was extended to predict drug interactions in paediatrics.

Scaling drug doses from adults to children is far from a straightforward process (Johnson, 2008). Both population pharmacokinetic and PBPK approaches have been used independently or in combination to guide drug dosing in paediatric patients (Johnson, 2005). A population pharmacokinetic model incorporating body weight as a primary covariate with an allometric exponent, e.g. ¾ for clearance, often does not perform well in infants and young children due to maturation of drug eliminating processes (Germovsek et al., 2017). In most cases, the predictions are improved by employing a sigmoidal ontogeny function driven by postmenstrual age (Anderson and Holford, 2011). However, the maturation half-life and Hill coefficient which parameterise the function vary across different drugs (Holford et al., 2013; Germovsek et al., 2017) and thus, sufficient number of individuals with age around the maturation half-life is necessary for precise parameter estimation. PBPK modeling and simulation offers an alternative approach to evaluate an optimal dosing regimen in the paediatric population. It integrates drug-specific inputs and system-related parameters, the latter of which encompass developmental changes in physiology and maturational rates of drug-metabolising enzymes and proteins involved in drug disposition (Maharaj and Edginton, 2014). This approach enables extrapolation from adults or between age groups within paediatric populations and increases the mechanistic understanding of potential sources of interindividual variability in systemic exposure to a drug.

The ontogeny profiles of key CYP enzymes responsible for imatinib metabolism (**Figure 2**) are based on a meta-analysis of *in vitro* CYP activity in post-mortem livers of donors from different ages (Johnson et al., 2006). The maturation functions tend to underestimate the apparent clearance of CYP3A substrates in neonates and infants (Salem et al., 2014). Two *in vivo*-derived algorithms have been proposed to improve the prediction (Salem et al., 2014; Upreti and Wahlstrom, 2016). The Upreti and Wahlstrom model for CYP3A4 maturation has been shown to perform better with less underprediction of clearance (Johnson et al., 2019). However, as expected, the PBPK simulations that implemented this ontogeny for children older than 2 years of age yielded a similar result to that of *in vitro* maturation function (results not shown). Therefore, the latter, which is incorporated in the Simcyp Simulator (version 17) by default, was utilized throughout the simulations. Developmental changes in organ size, particularly liver volume were also incorporated in the PBPK model. Liver volume was most parsimoniously described by a nonlinear regression against BSA as shown in Eq. 6 (Johnson et al., 2005). Interestingly, this equation was in concordance with an allometric weight model with an exponent of ¾ in estimating liver volume from infants to adolescents (Fanta et al., 2007). The correlation between liver volume and BSA alone was superior than that with other covariates (Johnson et al., 2005), in agreement with the findings of a nonlinear mixed effect modeling approach (Small et al., 2017). All the ontogeny equations used in the current study were driven by postnatal age. Postmenstrual age is more useful if preterm neonates are included in PBPK simulations (Abduljalil et al., 2019; Germovsek et al., 2019).

It is noteworthy that the PBPK models may overestimate clinically observed peak plasma concentrations (C_max_) since they report predicted concentrations at the central venous compartment rather than the peripheral vein from which blood (plasma) samples were collected. This is particularly important for intravenous (i.v.) administration routes where a substantial amount of drug is delivered to central venous compartment directly and equilibration to the peripheral venous sites may not be instantaneous (Musther et al., 2015). A PBPK model prediction of drug concentrations at a peripheral sampling site based on contributions from surrounding tissues (e.g., adipose, muscle, and skin) as proposed by Musther et al (2015) has proven to be useful to correct the PBPK predictions at initial time following i.v. administrations. As depicted in **Figure S1**, implementation of this strategy within the Simcyp Simulator improved the PBPK model predictions of C_max_ following a 1-h infusion of imatinib (100 mg) (Peng et al., 2004) compared to that of central venous compartment (prediction differences of imatinib C_max_ of 0.99 vs. 1.42). Prediction differences for other pharmacokinetic parameters of imatinib were similar between the two strategies (results not shown). Conversely, the peripheral sampling site model has little to no effect on PBPK predictions of C_max_ of imatinib given orally (results not shown). Unlike i.v. administration over a short period of time, oral administrations of drugs are likely to give sufficient time for central venous compartment (pooled venous return) and peripheral vein in the arm to equilibrate (Musther et al., 2015).

The observed interindividual variability of imatinib concentrations in children on day 1 appeared to be higher than that at steady-state from the corresponding patient cohort (**Figures 3F–I**). The reason for this trend was not clear, but may be related to a lower between individual variability in CYP3A4 activity due to the autoinhibition by imatinib following chronic exposure (Filppula et al., 2012; Filppula et al., 2013a). PBPK simulations also highlighted a higher interindividual variability of imatinib concentrations at a fixed daily dose compared to a BSA-normalized dosing regimen (**Figures 3M, N** vs. **3F–L**). A daily dose administered on a mg/m^2^ basis in paediatric populations is usually preferred to body weight-based and flat-fixed dosing regimens owing to more favorable pharmacokinetic variability, particularly over a wide age range (Bartelink et al., 2006; Hempel and Boos, 2007).

A clear exposure-response relationship for imatinib has not been established in younger patients with CML. Thus, the proposed targets in children and adolescents were based on the concentration known to be safe and efficacious in adults (C_min_ ranging from 1,000 to 3,200 ng/ml (Lankheet et al., 2017)). This was further supported by the similar biological and clinical features of CML observed in adult and younger patients (Barr, 2010), with only a slight difference, particularly a higher leukocyte count presented in the latter (Millot et al., 2005). Paediatric and adult patients also had comparable response and safety profiles (e.g., occurrence of grade 3/4 haematological toxicities and musculoskeletal adverse events) to an equivalent dose of imatinib (Millot et al., 2011). This was not the case for solid tumours harboring mutations in the gene that encodes tyrosine kinase KIT (e.g. GIST). Imatinib exerted minor anticancer activity in children with GIST compared to the adult cohort, despite similar systemic concentrations (Geoerger et al., 2009).

The observed trend of a higher interindividual variability of simulated C_min_ in children aged between 2 and 5 years and middle-aged adults compared to other age groups (**Figure 4**) was likely attributed to a higher variability within these age bands due to maturational changes of CYP enzymes that have not attained adult levels of expression (Johnson et al., 2006) and a reduction of total hepatic clearance related to a decrease of liver weight and scaling factor (e.g., microsomal protein per gram of liver/MPPGL) (Barter et al., 2007; Chetty et al., 2018), respectively.

PBPK simulations suggested a similar C_min_ following imatinib doses of 230 and 340 mg/m^2^/d in paediatrics and 400 and 600 mg/d in adult population, respectively *(p* > 0.01). This was in agreement with the finding in clinical studies in children with Ph+ leukaemias or GIST which indicated a similar systemic exposure of imatinib at daily doses of 230 and 340 mg/m^2^ compared to those of adult patients treated with 400 and 600 mg/d of imatinib, respectively (Champagne et al., 2004; Geoerger et al., 2009). The C_min_ target of 1,000 ng/ml was predicted to be attainable by a 230 mg/m^2^/d dose in paediatric age groups (similar to an adult dose of 400 mg/d), albeit with a large subset of the population below the target. Due to this variability, a higher dose of 340 mg/m^2^/d (corresponds to an adult dose of 600 mg/d) might be needed. This was in line with the recommendation for the treatment of CML in children with the recommended initial doses of 260–300 mg/m^2^/d and 400 mg/m^2^/d for chronic and accelerated phases, respectively (de la Fuente et al., 2014).

There was a good agreement between PBPK model prediction and clinically observed changes in imatinib concentrations due to the coadministration of carbamazepine in adult and paediatric populations (**Figure 6**). It should be noted that clinical pharmacokinetic data in the latter came from one Japanese paediatric patient (a case study) (Taguchi et al., 2014). PBPK simulations in paediatrics refer to European ancestry, from which the ontogeny functions for drug-metabolising enzymes and AAG were derived (Johnson et al., 2006). However, our previous simulation study suggested little to no difference in imatinib pharmacokinetic between people from Japanese and European ancestry (unpublished).

Clinical drug-drug interaction (DDI) data in adults may not be suitable for extrapolation across all paediatric age bands (Salem et al., 2013b; Salerno et al., 2019). The magnitudes of enzyme-based DDI are dictated by the level of contribution (f_m_) and maturational rates of corresponding CYP enzymes (Salem et al., 2013a). In this study, a PBPK modeling approach was utilized to evaluate drug interactions with imatinib in paediatrics. The trend and extent of interactions between imatinib and CYP3A modulators (carbamazepine, rifampicin and ketoconazole) were predicted to be similar between paediatric and adult populations, despite a slight difference in the simulated means and interindividual variabilities (**Figure 7**). Imatinib inhibits its own CYP3A4-mediated metabolism following multiple-dosing regimen (Filppula et al., 2012). Thus, the effect of CYP3A modulators on imatinib metabolism was likely to be diminished following a long-term use of imatinib, as observed in a clinical interaction study between imatinib and ritonavir (van Erp et al., 2007). The extent of modulation by CYP3A inhibitors, either direct (reversible) or mechanism-based inhibitors, e.g., ketoconazole and ritonavir, respectively was predicted to be more affected following repeated-dose administration of imatinib, compared to that observed with CYP3A inducers (e.g., rifampicin and carbamazepine). This was due to limited residual CYP3A activity which can further be inhibited in the former. Since imatinib undergoes little to no metabolism in the enterocytes (Barratt and Somogyi, 2017), inducers of CYP3A confined to intestinal enzymes (e.g., hyperforin in St John’s wort) are unlikely to affect steady-state CL/F of imatinib (Adiwidjaja et al., 2019).

The limitation of this study is a lack of specific maturation functions for children with cancer implemented in the PBPK model. The trend of developmental changes in organ size, CYP enzymes and plasma proteins observed in healthy children may not hold true for the paediatric cancer population (Thai et al., 2015). A further limitation to this study is the exclusion of children less than 2 years of age from the simulations (**Figures 4** and **7**) due to a paucity of clinical pharmacokinetic data for this age group (CML is exceptionally rare in very young children (de la Fuente et al., 2014)). Moreover, there is a high uncertainty in the maturation pattern of CYP3A4 in this challenging age group (Johnson et al., 2019), which is further complicated by the potential presence of CYP3A7 enzyme. The latter is absent in adults, but expressed at a high level during foetal life and decreases progressively throughout the first 2 years after birth (Allegaert and van den Anker, 2019). A further study to elucidate CYP3A7 contribution to imatinib metabolism is necessary in order to perform a PBPK prediction with confidence in children less than 2 years.

In conclusion, a PBPK model for imatinib was successfully developed in adults and extrapolated to the paediatric population. The PBPK model was able to describe clinical pharmacokinetic data from published studies observed in adults, children and adolescents. PBPK simulation suggested an optimal dosing regimen range for imatinib of 230–340 mg/m^2^/d in paediatrics, in concordance with the recommended initial dose for treatment of childhood CML. The simulations also highlighted that children and adults being treated with imatinib have similar vulnerability to drug interactions that modulate drug metabolising enzyme activity. These findings suggest that at steady-state, imatinib is more susceptible to hepatic induction compared to inhibition of CYP3A enzymes.

## Data Availability Statement

All datasets generated for this study are included in the article/**Supplementary Material**.

## Author Contributions

JA, AB, and AM wrote the manuscript, designed the research, and contributed to the interpretation. JA performed the simulations and analyzed the data.

## Conflict of Interest

The authors declare that the research was conducted in the absence of any commercial or financial relationships that could be construed as a potential conflict of interest.
