# Supplementary material for: Implementation of a Physiologically Based Pharmacokinetic Modeling Approach to Guide Optimal Dosing Regimens for Imatinib and Potential Drug Interactions in Paediatrics
Source: Front Pharmacol. 2020 Jan 30;10:1672. doi: 10.3389/fphar.2019.01672 (PMC7002565; doi:10.3389/fphar.2019.01672)
Supplement: Supplementary file 1 [file DataSheet_1.docx]

**Supplementary materials**

**Table S1** Comparison of PBPK model prediction and clinical pharmacokinetic parameters of imatinib in an interaction study with ritonavir in adult patients ^a)^

|  | **C_ss,max_ (mg/L)** | | | **AUC_24_ (mg.h/L)** | | | **CL/F (L/h)** | | |
| --- | --- | --- | --- | --- | --- | --- | --- | --- | --- |
|  | **Imatinib alone** | **With ritonavir** | **Ratio** | **Imatinib alone** | **With ritonavir** | **Ratio** | **Imatinib alone** | **With ritonavir** | **Ratio** |
| **Clinically-observed values** | 2.9  (2.3 - 3.7) | 2.5  (1.9 - 3.2) | 0.86 | 42.6  (33.0 - 54.9) | 41.2  (32.1 - 53.1) | 0.97 | 9.4  (7.3 - 12.1) | 9.7  (7.5 - 12.5) | 1.03 |
| **PBPK model predicted values** | | | | | | | | | |
| Without an MBI of CYP3A4 nor compensatory clearance in the PBPK model of imatinib | | | | | | | | | |
| PBPK prediction | 2.5 | 4.3 | 1.72 | 34.6 | 75.2 | 2.17 | 11.6 | 5.3 | 0.46 |
| **Prediction fold-difference** ^b)^ | 0.86 | 1.72 | 2.00 | 0.81 | 1.83 | 2.24 | 1.23 | 0.55 | 0.45 |
| Incorporating an MBI of CYP3A4, but not a compensatory clearance in the PBPK model of imatinib | | | | | | | | | |
| PBPK prediction | 3.6 | 4.0 | 1.11 | 56.2 | 65.0 | 1.16 | 7.1 | 6.2 | 0.87 |
| **Prediction fold-difference** | 1.24 | 1.60 | 1.29 | 1.32 | 1.58 | 1.20 | 0.76 | 0.64 | 0.84 |
| Incorporating both MBI of CYP3A4 and compensatory clearance in the PBPK model of imatinib | | | | | | | | | |
| PBPK prediction | 2.7 | 2.9 | 1.07 | 37.7 | 42.3 | 1.12 | 10.6 | 9.5 | 0.89 |
| **Prediction fold-difference** | **0.93** | **1.16** | **1.24** | **0.88** | **1.03** | **1.15** | **1.13** | **0.98** | **0.86** |

AUC_24_, area under the plasma concentration-time curve during 24 h after dose; CL/F, apparent clearance; C_ss,max_, peak plasma concentration at steady-state; CYP, cytochrome P450 enzyme; MBI, mechanism-based inhibition.

^a)^ A clinical interaction study in patients with gastrointestinal stromal tumours (GIST; 5 male, 6 female; age 51 – 79 years) receiving 400 mg daily dose of imatinib for at least 2 months. Ritonavir (600 mg/d) was given concomitantly with imatinib for 3 d (van Erp et al., 2007).

^b)^ Prediction-fold differences were expressed as the ratio of PBPK model prediction to clinically-observed values.

**Table S2** PBPK model predictions and clinically-observed pharmacokinetic parameters of carbamazepine and its active metabolite in the presence and absence of CYP2C8 induction

| **Parameter** | **Clinically-observed values** | **Without CYP2C8 induction** | | **Accounting for CYP2C8 induction** | |
| --- | --- | --- | --- | --- | --- |
|  |  | **PBPK model prediction ^a)^** | **Prediction fold-difference** | **PBPK model prediction** | **Prediction fold-difference** |
| Carbamazepine (300 mg bid) at steady-state (Carlsson et al., 2005) | | | | | |
| **Carbamazepine** | | | | | |
| CL/F (L/h) | 3.6 ^b)^ | 3.2 | **0.89** | 3.8 | **1.06** |
| CV of CL/F (%) | 52 ^c)^ | 53 |  | 54 |  |
| Carbamazepine (9.5 mg/kg bid) at steady-state (Eeg-Olofsson et al., 1990) | | | | | |
| **Carbamazepine** | | | | | |
| C_ss,max_ (µmol/L) | 39.8 ± 10.0 | 45.6 | **1.15** | 40.2 | **1.01** |
| C_min_ (µmol/L) | 21.5 ± 5.8 | 26.9 | **1.25** | 19.0 | **0.88** |
| AUC_24_ (µmol.h/L) | 762.5 ± 163.2 | 870.3 | **1.14** | 742.3 | **0.97** |
| **Carbamazepine-10,11-epoxide** | | | | | |
| C_ss,max_ (µmol/L) | 6.0 ± 2.3 | 6.9 | **1.15** | 5.5 | **0.92** |
| C_min_ (µmol/L) | 4.0 ± 1.6 | 5.8 | **1.45** | 4.5 | **1.13** |
| AUC_24_ (µmol.h/L) | 138.0 ± 48.9 | 155.3 | **1.13** | 121.4 | **0.88** |

AUC_24_, area under the plasma concentration-time curve during 24 h after dose; bid, twice daily; C_min_, trough concentration; C_ss,max_, peak plasma concentration at steady-state; CL/F, apparent clearance; CV, coefficient of variation.

^a)^ Reported as geometric mean values of PBPK model prediction.

^b)^ Typical population value.

^c)^ Based on ω (standard deviation of eta, inter-individual variability) of apparent clearance.

**
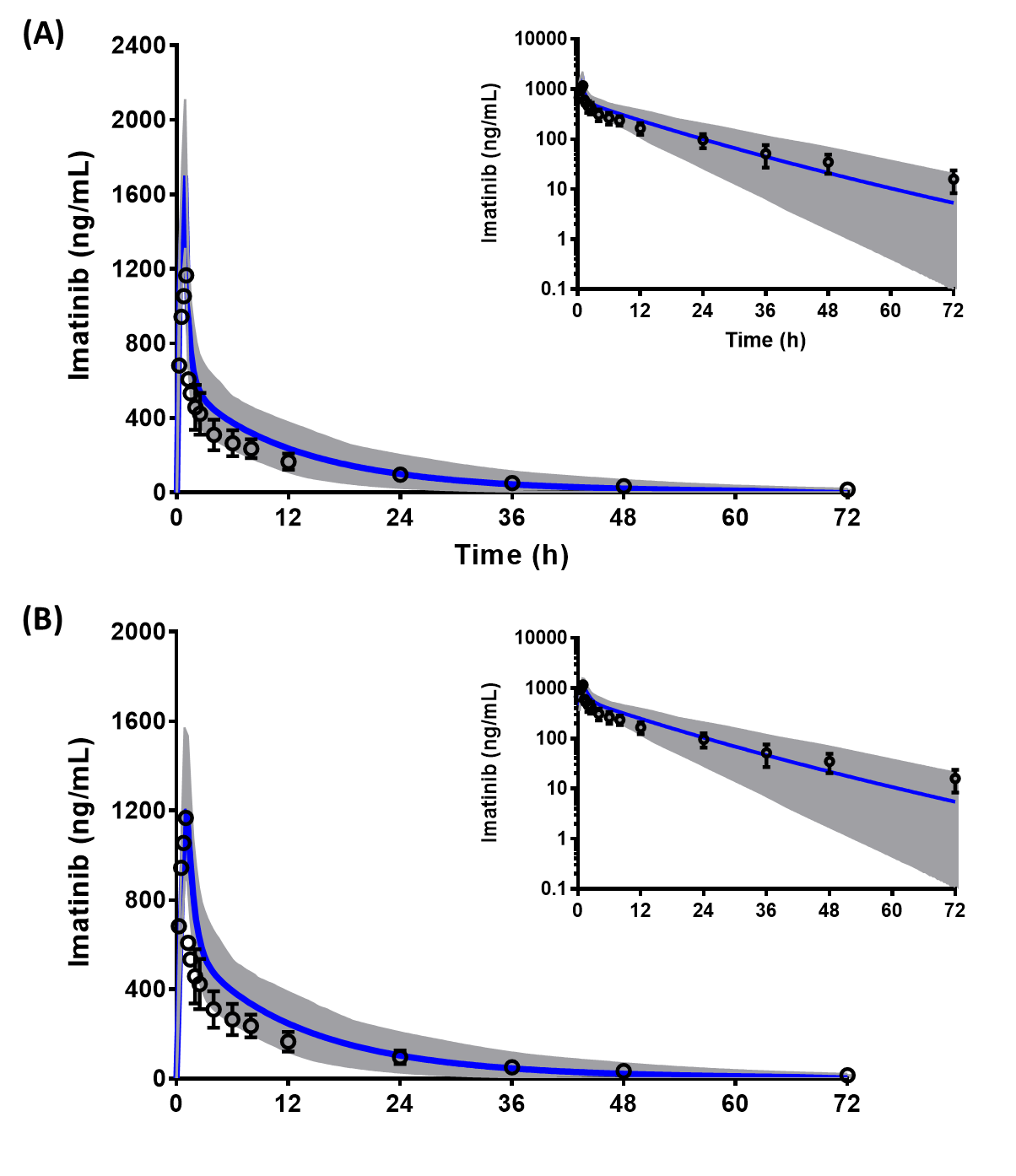
**

**Figure S1** Clinically-observed concentrations of imatinib (dot: mean, error bar: standard deviation) overlaid with mean (blue line) and 5^th^ to 95^th^ percentiles (grey area) of the PBPK model predicted concentrations using central venous (A) and peripheral sampling site compartments (B). The predictions are depicted in linear scale with the corresponding semi-logarithmic plots as insets. Imatinib was given as a single 1-hour intravenous infusion to healthy people (n = 12, 2 females, aged 40–58 years) (Peng et al., 2004).

**References**

Carlsson, K.C., Hoem, N.O., Glauser, T., and Vinks, A.A. (2005). Development of a population pharmacokinetic model for carbamazepine based on sparse therapeutic monitoring data from pediatric patients with epilepsy. *Clin Ther* 27(5)**,** 618-626.

Eeg-Olofsson, O., Nilsson, H.L., Tonnby, B., Arvidsson, J., Grahn, P.A., Gylje, H., et al. (1990). Diurnal variation of carbamazepine and carbamazepine-10,11-epoxide in plasma and saliva in children with epilepsy: a comparison between conventional and slow-release formulations. *J Child Neurol* 5(2)**,** 159-165.

Peng, B., Dutreix, C., Mehring, G., Hayes, M.J., Ben-Am, M., Seiberling, M., et al. (2004). Absolute bioavailability of imatinib (Glivec) orally versus intravenous infusion. *J Clin Pharmacol* 44(2)**,** 158-162.

van Erp, N.P., Gelderblom, H., Karlsson, M.O., Li, J., Zhao, M., Ouwerkerk, J., et al. (2007). Influence of CYP3A4 inhibition on the steady-state pharmacokinetics of imatinib. *Clin Cancer Res* 13(24)**,** 7394-7400.
